# Therapeutic role of low molecular weight heparin and fat-soluble vitamins in pregnancy-associated prethrombotic conditions

**DOI:** 10.3389/fmed.2026.1883377

**Published:** 2026-08-19

**Authors:** He Yanhong, He Cuiyi, Yu Ruiling, Huang Guowei

**Affiliations:** Department of Obstetrics, Shunde Hospital Affiliated to Jinan University, Foshan, Guangdong, China

**Keywords:** endothelial dysfunction, fat-soluble vitamins, hypercoagulability, immunothrombosis, low molecular weight heparin (LMWH), maternal–fetal health, placental insufficiency, preeclampsia

## Abstract

Pregnancy is a physiological hypercoagulable state associated with an increased risk of venous thromboembolism (VTE) and other prethrombotic conditions that contribute significantly to maternal and fetal morbidity and mortality. While low molecular weight heparin (LMWH) remains the cornerstone of prophylaxis and treatment due to its efficacy and safety profile, emerging evidence suggests that thrombosis in pregnancy is a multifactorial process involving endothelial dysfunction, inflammation, oxidative stress, and impaired placental development. These interconnected pathways highlight the limitations of anticoagulation alone in addressing the full spectrum of disease mechanisms. Fat-soluble vitamins, including vitamins D, K, E, and A, play critical roles in regulating vascular integrity, immune responses, oxidative balance, and coagulation pathways. Deficiencies or imbalances in these vitamins have been associated with adverse pregnancy outcomes such as preeclampsia, intrauterine growth restriction, recurrent pregnancy loss, and preterm birth. Mechanistically, these vitamins modulate upstream processes that contribute to thrombotic risk, complementing the downstream anticoagulant effects of LMWH. This review synthesizes current evidence on the pathophysiology of pregnancy-associated prethrombotic conditions, the therapeutic role of LMWH, and the biological functions of fat-soluble vitamins in maternal–fetal health. Furthermore, it proposes a synergistic model in which LMWH and fat-soluble vitamins act through complementary mechanisms to target both the causes and consequences of thrombosis. Clinical evidence supporting the independent roles of these interventions is discussed, and a conceptual model is presented. Despite a strong mechanistic rationale, clinical data evaluating combined LMWH and vitamin-based strategies remain limited. Future research should focus on randomized controlled trials, biomarker-guided approaches, and precision medicine strategies to optimize treatment. The integration of anticoagulant therapy with targeted nutritional modulation represents a promising avenue for improving pregnancy outcomes and advancing the management of thrombotic disorders in maternal–fetal medicine.

## 1 Introduction

Pregnancy is a unique physiological state characterized by profound hematological, immunological, and vascular adaptations that are essential for maintaining maternal–fetal homeostasis. Among these changes, a shift toward a hypercoagulable state is particularly prominent, representing an evolutionary mechanism designed to minimize hemorrhage during miscarriage and childbirth. This state is driven by increased levels of procoagulant factors, reduced fibrinolytic activity, and alterations in endogenous anticoagulant pathways, collectively predisposing pregnant women to thrombotic complications (1, 2). While these changes are physiologically protective, they also significantly elevate the risk of venous thromboembolism (VTE), making it one of the leading causes of maternal morbidity and mortality worldwide.

Beyond overt thrombotic events such as deep vein thrombosis and pulmonary embolism, pregnancy-associated prethrombotic conditions encompass a broader spectrum of pathophysiological disturbances. These include thrombophilia, endothelial dysfunction, and placental vascular abnormalities, all of which contribute to adverse obstetric outcomes (3). Notably, conditions such as preeclampsia, intrauterine growth restriction, recurrent pregnancy loss, and placental abruption have been increasingly linked to microthrombotic processes and impaired placental perfusion. The interplay between coagulation activation, inflammation, and endothelial injury highlights the concept of “immunothrombosis” as a central mechanism underlying these complications (4, 5).

Low molecular weight heparin (LMWH) has emerged as a cornerstone in the management of pregnancy-associated thrombotic disorders. Owing to its favorable pharmacokinetic profile and inability to cross the placenta, LMWH is considered safe for both the mother and fetus. Its primary anticoagulant effect is mediated through inhibition of factor Xa; however, accumulating evidence suggests that LMWH also exerts pleiotropic effects, including anti-inflammatory activity, modulation of endothelial function, and improvement of placental microcirculation. These properties extend its therapeutic relevance beyond classical thrombosis prevention, positioning LMWH as a potential modulator of placental health and pregnancy outcomes (6–8).

In parallel, growing attention has been directed toward the role of micronutrients, particularly fat-soluble vitamins, in the regulation of coagulation, inflammation, and vascular integrity during pregnancy. Vitamin D, for instance, has been implicated in immune modulation and endothelial function, with deficiency associated with increased risks of preeclampsia, gestational diabetes, and adverse fetal outcomes. Vitamin K plays a critical role in the activation of coagulation factors, maintaining the delicate balance between thrombosis and bleeding, while also contributing to vascular health. Additionally, vitamins A and E are involved in oxidative stress regulation, immune responses, and placental development, all of which are crucial for maintaining a healthy pregnancy (9–11).

Despite the established individual roles of LMWH and fat-soluble vitamins, their combined or complementary effects in managing pregnancy-associated prethrombotic conditions remain underexplored. Understanding how anticoagulant therapy and micronutrient status intersect may provide new insights into more effective and personalized therapeutic strategies. Therefore, this review aims to comprehensively examine the pathophysiology of pregnancy-associated prethrombotic states and to evaluate the therapeutic roles of LMWH and fat-soluble vitamins, with a particular focus on their mechanisms of action, clinical applications, and potential synergistic effects in improving maternal and fetal outcomes.

This narrative review was developed through a comprehensive literature search of PubMed, Web of Science, Scopus, and Google Scholar, focusing primarily on studies published between 2010 and 2025. Search terms included combinations of pregnancy, pregnancy-associated prethrombotic state, venous thromboembolism, low-molecular-weight heparin, vitamin A, vitamin D, vitamin E, vitamin K, immunothrombosis, placental dysfunction, preeclampsia, and recurrent pregnancy loss. Original research articles, randomized controlled trials, observational studies, systematic reviews, meta-analyses, clinical guidelines, and relevant experimental studies were included. Human studies were prioritized, while animal studies were incorporated when they provided important mechanistic insights into the pathophysiology of pregnancy-associated prethrombotic conditions or the biological effects of LMWH and fat-soluble vitamins. Earlier landmark studies published before 2010 were also included when considered essential for historical context or foundational knowledge.

## 2 Pathophysiology of pregnancy-associated prethrombotic conditions

**FIGURE 1:**
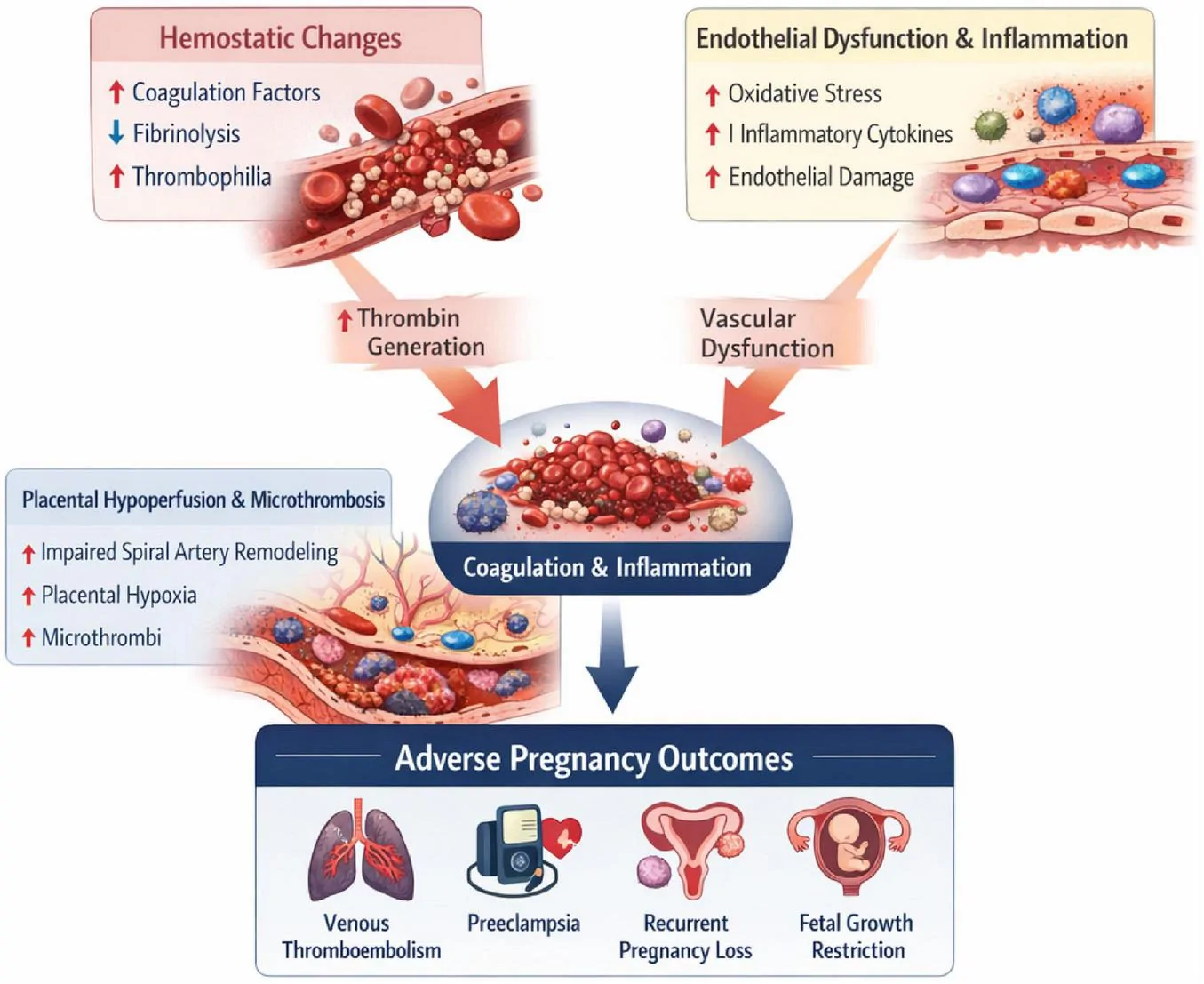
Mechanisms of pregnancy-associated hypercoagulability and venous thromboembolism (VTE). Schematic illustration of physiological changes during pregnancy, including increased coagulation factors, reduced fibrinolysis, endothelial dysfunction, and venous stasis, which collectively promote thrombus formation and elevate the risk of VTE.

Pregnancy is associated with dynamic and tightly regulated changes in the hemostatic system, resulting in a physiological shift toward hypercoagulability. This transition reflects a coordinated adaptation involving the coagulation cascade, fibrinolytic pathways, vascular endothelium, and immune system. While essential for preventing excessive bleeding during delivery, these changes also predispose pregnant women to a spectrum of prethrombotic conditions, ranging from subclinical microthrombosis to clinically significant thromboembolic events (12–15) (5). As illustrated in Figure 1, pregnancy-induced hormonal and vascular changes lead to endothelial dysfunction, hypercoagulability, and impaired blood flow, collectively contributing to thrombus formation and adverse maternal outcomes.

### 2.1 Hemostatic changes during pregnancy

One of the hallmarks of pregnancy is the upregulation of procoagulant factors, including factors VII, VIII, X, and fibrinogen, alongside increased levels of von Willebrand factor. Concurrently, natural anticoagulant mechanisms are attenuated, particularly through a reduction in free protein S levels and increased resistance to activated protein C (16–18). In parallel, fibrinolytic activity is suppressed due to elevated concentrations of plasminogen activator inhibitors (PAI-1 and placental-derived PAI-2) (19). Collectively, these alterations enhance thrombin generation and promote fibrin formation, establishing a prothrombotic milieu early in gestation and persisting into the postpartum period. Importantly, these hemostatic adaptations are not uniform across all individuals. Women with inherited or acquired thrombophilia, such as factor V Leiden mutation, prothrombin gene mutation, or antiphospholipid syndrome, exhibit an exaggerated procoagulant response. This amplifies the risk of venous thromboembolism and contributes to adverse pregnancy outcomes, highlighting the interplay between baseline risk factors and pregnancy-induced changes (20, 21).

### 2.2 Endothelial dysfunction and inflammation

Beyond coagulation factors, endothelial dysfunction plays a central role in the development of pregnancy-associated prethrombotic conditions. The vascular endothelium, which normally maintains an antithrombotic surface, becomes activated under the influence of oxidative stress, inflammatory cytokines, and circulating antiangiogenic factors (22–24). This activation results in increased expression of adhesion molecules, tissue factor, and proinflammatory mediators, promoting leukocyte recruitment and platelet aggregation. Inflammation further exacerbates this process by linking immune activation to coagulation pathways—a phenomenon often referred to as immunothrombosis. In pregnancy complications such as preeclampsia, elevated levels of antiangiogenic factors (e.g., soluble FMS-like tyrosine kinase-1 and soluble endoglin) disrupt endothelial integrity and nitric oxide signaling. This leads to vasoconstriction, increased vascular permeability, and enhanced thrombotic tendency. The convergence of inflammation and coagulation thus represents a key mechanistic axis in the pathogenesis of pregnancy-related vascular disorders (25–28).

### 2.3 Placental hypoperfusion and microthrombosis

The placenta is particularly vulnerable to thrombotic and vascular disturbances due to its unique structure and high metabolic demand. Abnormal placentation, characterized by inadequate remodeling of spiral arteries, results in reduced uteroplacental blood flow and chronic hypoxia. This hypoxic environment stimulates the release of proinflammatory and antiangiogenic mediators, further aggravating endothelial dysfunction and promoting localized coagulation activation (29–31). Microthrombi formation within placental vessels impairs nutrient and oxygen exchange, contributing to a range of obstetric complications, including intrauterine growth restriction, recurrent pregnancy loss, and placental abruption. In severe cases, widespread endothelial damage and systemic coagulation activation culminate in preeclampsia, a condition marked by hypertension and multi-organ involvement (32, 33).

## 3 Low molecular weight heparin (LMWH): mechanisms and therapeutic roles

**FIGURE 2:**
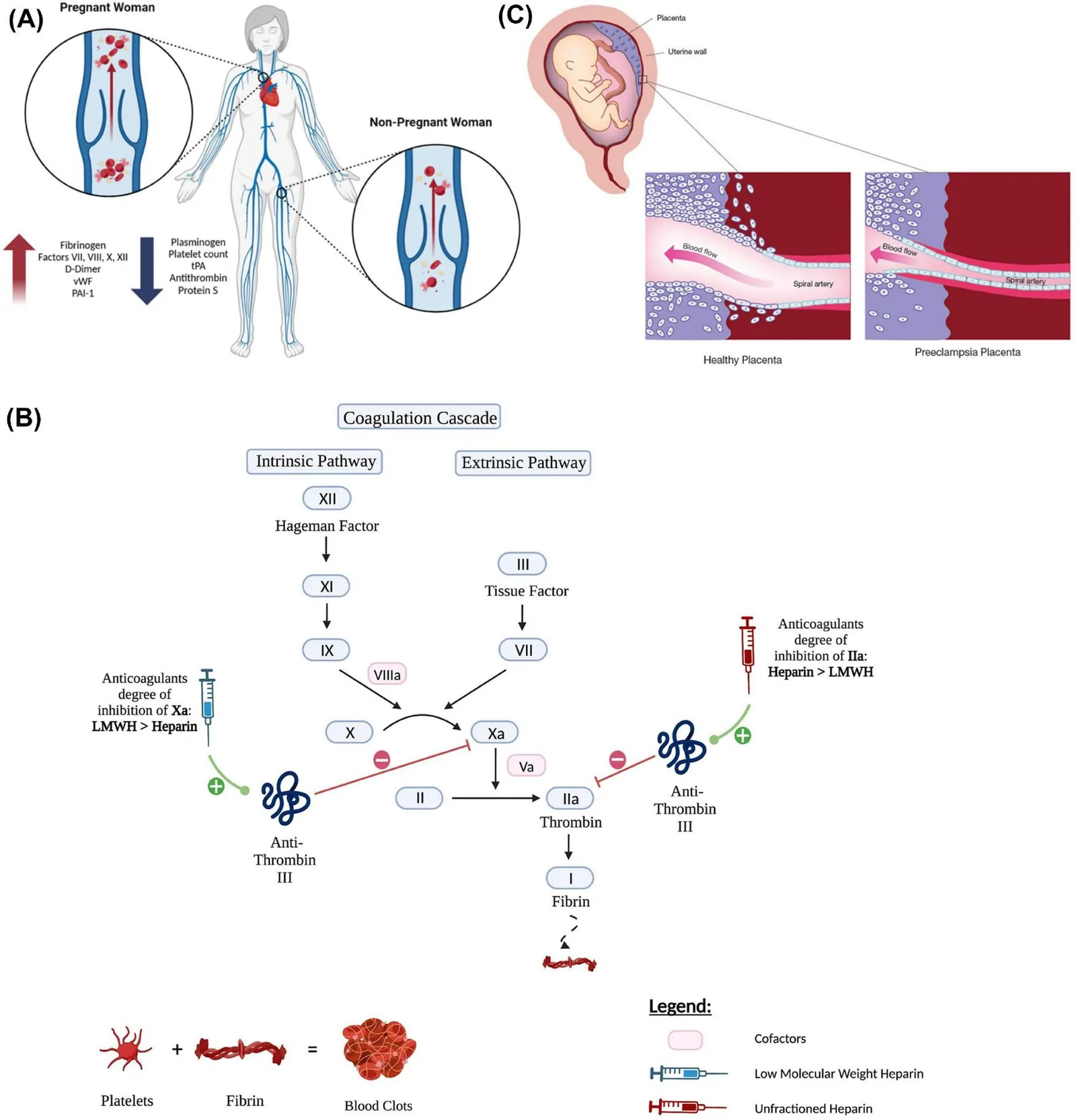
Mechanistic and clinical overview of low-molecular-weight heparin (LMWH) in pregnancy. **(A)** Pregnancy induces a hypercoagulable state characterized by increased coagulation factors and reduced fibrinolysis, leading to elevated risk of venous thromboembolism (VTE) compared with non-pregnant women. **(B)** Schematic of the coagulation cascade showing intrinsic and extrinsic pathways converging at factor Xa and thrombin generation. LMWH enhances antithrombin III activity, preferentially inhibiting factor Xa and reducing thrombin formation, thereby limiting fibrin clot development (with greater anti-Xa selectivity than unfractionated heparin). **(C)** Impact of coagulation imbalance on placental vasculature. Normal pregnancy is associated with adequate spiral artery remodeling and blood flow, whereas dysregulated coagulation contributes to placental insufficiency and disorders such as preeclampsia. LMWH may improve placental microcirculation by reducing thrombotic burden and supporting vascular function.

Low molecular weight heparin (LMWH) has become the cornerstone of anticoagulant therapy in pregnancy-associated prethrombotic conditions due to its well-established efficacy, safety profile, and favorable pharmacological characteristics. In the context of pregnancy, where both maternal and fetal safety must be carefully balanced, LMWH is preferred over other anticoagulants because it does not cross the placenta and therefore does not exert direct teratogenic or anticoagulant effects on the fetus. This distinguishes it from vitamin K antagonists such as warfarin, which are associated with embryopathy and fetal bleeding (34–35). The increasing use of LMWH in obstetric practice reflects its ability to effectively reduce thrombotic risk while maintaining a high degree of safety, making it the first-line therapy for both prophylaxis and treatment of venous thromboembolism (VTE) during pregnancy and the postpartum period. As illustrated in Figure 2, pregnancy induces a hypercoagulable state, LMWH selectively inhibits factor Xa within the coagulation cascade, and these effects translate into improved placental perfusion and reduced thrombotic complications.

### 3.1 Pharmacological properties of LMWH

LMWH is produced through the depolymerization of unfractionated heparin, resulting in shorter polysaccharide chains with a more predictable anticoagulant profile. Its mechanism of action is primarily mediated through binding to antithrombin, inducing a conformational change that enhances the inhibition of activated factor Xa and, to a lesser extent, thrombin (factor IIa) (36, 37). This selective inhibition reduces thrombin generation and fibrin formation, thereby limiting clot propagation. Compared to unfractionated heparin, LMWH demonstrates improved bioavailability following subcutaneous administration, a longer plasma half-life, and reduced nonspecific binding to plasma proteins and endothelial cells. These features contribute to a more stable and predictable anticoagulant response, often eliminating the need for routine laboratory monitoring. Additionally, LMWH is associated with a lower incidence of adverse effects such as osteoporosis and heparin-induced thrombocytopenia (HIT), which are more commonly observed with unfractionated heparin. The pharmacokinetic stability of LMWH is particularly advantageous in pregnancy, where physiological changes such as increased plasma volume and renal clearance can complicate drug dosing and efficacy (38).

### 3.2 Antithrombotic and anti-inflammatory effects

Beyond its classical role as an anticoagulant, LMWH exerts a range of biological effects that extend into the modulation of inflammation and vascular function. Increasing evidence suggests that LMWH can interact with endothelial cells, cytokines, and growth factors, thereby influencing processes that are critical for placental development and function. For instance, LMWH has been shown to reduce the expression of proinflammatory cytokines and adhesion molecules, limiting leukocyte recruitment and endothelial activation. This is particularly relevant in pregnancy complications such as preeclampsia, where inflammation and endothelial dysfunction are central pathogenic features. Furthermore, LMWH may enhance placental perfusion by preventing the formation of microthrombi within the uteroplacental circulation and improving microvascular blood flow (39–41). As illustrated in Figure 2, LMWH acts not only by inhibiting factor Xa to reduce thrombin generation but also by preserving endothelial integrity and mitigating inflammatory signaling, thereby contributing to improved placental function and pregnancy outcomes. These pleiotropic effects highlight the broader therapeutic potential of LMWH beyond simple anticoagulation.

### 3.3 Clinical applications in pregnancy

Clinically, LMWH is widely utilized across a spectrum of pregnancy-associated conditions characterized by increased thrombotic risk. It is the standard of care for the treatment and prevention of VTE, including deep vein thrombosis and pulmonary embolism, which remain major contributors to maternal morbidity and mortality. In addition, LMWH is frequently prescribed for women with inherited or acquired thrombophilia, particularly those with a history of thrombotic events or recurrent pregnancy loss (42–44). Emerging evidence also supports its use in women with polycystic ovary syndrome (PCOS), a condition associated with a prothrombotic state and impaired implantation. In such populations, LMWH therapy during early pregnancy has been shown to significantly reduce the rate of early pregnancy loss compared to untreated controls. The beneficial effects observed in these clinical settings are thought to arise not only from anticoagulation but also from improved placental vascularization and modulation of the maternal immune response. The clinical applications of LMWH in pregnancy-associated prethrombotic conditions, along with their underlying mechanisms and reported outcomes, are summarized in Table 1 (45–49).

**TABLE 1:** Clinical applications of LMWH in pregnancy-associated prethrombotic conditions.

Condition	Pathophysiology/risk basis	Role of LMWH	Key clinical evidence from sources	Clinical outcome
Venous thromboembolism (VTE)	Pregnancy-induced hypercoagulability, venous stasis, endothelial changes	First-line anticoagulant for treatment and prophylaxis	Pregnancy increases VTE risk ∼4–5 × ; anticoagulation improves outcomes	↓ DVT, ↓ PE, ↓ maternal mortality
Thrombophilia (inherited/acquired)	Exaggerated coagulation response (e.g., Factor V Leiden, APS)	Prevents thrombosis and pregnancy complications	Thrombophilia present in up to 20–50% of pregnancy-associated VTE cases	↓ recurrent VTE, improved pregnancy outcomes
Recurrent pregnancy loss (RPL)	Placental microthrombosis, impaired implantation	Improves uteroplacental blood flow	LMWH reduces early pregnancy loss rates (e.g., PCOS-related EPL reduction)	↓ Miscarriage rate
Polycystic ovary syndrome (PCOS)	Prothrombotic and inflammatory state affecting implantation	Adjunct therapy during early pregnancy	LMWH significantly reduced early pregnancy loss compared to control	↑ Pregnancy success rate
Preeclampsia/placental insufficiency	Endothelial dysfunction, microvascular thrombosis, poor placental perfusion	Improves placental microcirculation and reduces thrombosis	Placental dysfunction linked to coagulation imbalance and vascular injury	↓ Severity of placental complications (supportive evidence)
Postpartum thrombosis risk	Peak hypercoagulability ++ vascular injury after delivery	Extended prophylaxis in high-risk patients	Postpartum VTE risk significantly elevated (up to ∼20-fold or higher)	↓ Postpartum VTE incidence
High-risk patients (history of VTE)	Recurrence risk due to prior thrombotic events	Secondary prevention	Recurrence risk reduced with anticoagulation therapy	↓ Recurrent VTE

### 3.4 Limitations and adverse effects

Despite its advantages, LMWH therapy is associated with several limitations that must be considered in clinical practice. Bleeding remains the most significant adverse effect, ranging from minor injection-site hematomas to more serious complications such as internal hemorrhage and rectus sheath hematoma. Although the overall risk of major bleeding is relatively low, it necessitates careful patient selection and monitoring, particularly in individuals with additional risk factors. Another important complication is heparin-induced thrombocytopenia (HIT), a rare but potentially life-threatening immune-mediated condition characterized by a paradoxical increase in thrombotic risk despite thrombocytopenia (50). The diagnosis and management of HIT in pregnancy are particularly challenging due to limited alternative anticoagulant options and the need to balance maternal and fetal safety. Furthermore, practical issues such as the requirement for subcutaneous injections, patient compliance, and cost may limit long-term use. Physiological changes during pregnancy, including increased renal clearance and altered drug distribution, also raise questions regarding optimal dosing strategies, which remain an area of ongoing investigation (51–53).

## 4 Fat-soluble vitamins in pregnancy-associated thrombotic disorders

Fat-soluble vitamins, including vitamins D, K, E, and A, play critical and multifaceted roles in the regulation of coagulation, immune responses, oxidative stress, and vascular function during pregnancy. These biological processes are closely interconnected with the pathogenesis of pregnancy-associated prethrombotic conditions, which are increasingly recognized as disorders of vascular dysfunction and immunothrombosis rather than isolated coagulation abnormalities. While low molecular weight heparin (LMWH) exerts a direct anticoagulant effect by targeting the coagulation cascade, fat-soluble vitamins modulate upstream pathways that influence endothelial integrity, inflammatory signaling, and placental development. Consequently, disturbances in vitamin homeostasis may exacerbate the hypercoagulable state of pregnancy and contribute to adverse outcomes such as preeclampsia, intrauterine growth restriction, recurrent pregnancy loss, and preterm birth. Understanding the mechanistic roles of these vitamins provides important insight into their potential as complementary modulators of thrombotic risk and placental health (54–57).

Among these, vitamin D has emerged as a key regulator of immunothrombosis and vascular function during pregnancy. Acting through the vitamin D receptor, which is widely expressed in trophoblasts, endothelial cells, and immune cells, vitamin D influences gene expression related to inflammation, coagulation, and vascular tone. Adequate vitamin D levels support endothelial function by enhancing nitric oxide production and suppressing the expression of adhesion molecules, thereby limiting leukocyte adhesion and platelet activation. In addition, vitamin D modulates the balance between pro- and anti-inflammatory cytokines, reducing levels of tumor necrosis factor-α and interleukin-6 while promoting anti-inflammatory pathways (58, 59). Importantly, vitamin D deficiency has been associated with increased levels of plasminogen activator inhibitor-1, leading to reduced fibrinolysis and a more persistent prothrombotic state. These molecular alterations contribute to endothelial dysfunction and impaired placental perfusion, linking vitamin D deficiency to clinical conditions such as preeclampsia, fetal growth restriction, miscarriage, and preterm birth. Furthermore, vitamin D plays a role in trophoblast invasion and spiral artery remodeling, processes that are essential for establishing effective uteroplacental circulation. Together, these findings position vitamin D as a central modulator of the vascular and immune environment in pregnancy (54, 60–62).

Vitamin K, in contrast, occupies a more direct position within the coagulation system by serving as an essential cofactor for the γ-carboxylation of coagulation factors II, VII, IX, and X, as well as the natural anticoagulants protein C and protein S. This dual involvement underscores its role in maintaining hemostatic balance, which is particularly critical in pregnancy where both thrombotic and hemorrhagic risks are elevated. Disruption of vitamin K status can lead to impaired activation of these proteins, resulting in either excessive bleeding or dysregulated coagulation (63). In addition to its classical role in coagulation, vitamin K contributes to vascular health through vitamin K–dependent proteins such as matrix Gla protein, which regulates vascular calcification and maintains endothelial integrity. Although the direct contribution of vitamin K to the prevention of pregnancy-associated thrombosis is less clearly defined compared to LMWH, its central role in coagulation homeostasis makes it an important factor in preserving the delicate equilibrium between clot formation and resolution. Moreover, the interaction between vitamin K metabolism and anticoagulant therapies highlights the need for careful consideration of nutritional status in patients receiving pharmacological interventions (64, 65).

Vitamin E provides a distinct yet complementary mechanism through its potent antioxidant activity, which is particularly relevant in the context of pregnancy-related oxidative stress. Oxidative stress is a major contributor to endothelial dysfunction and is strongly implicated in the pathogenesis of preeclampsia and placental insufficiency. By scavenging reactive oxygen species and protecting lipid membranes from peroxidation, vitamin E helps maintain endothelial stability and supports normal vascular function (66–68). These antioxidant effects also extend to the regulation of platelet activity, as oxidative stress can enhance platelet aggregation and promote a procoagulant state. By mitigating these processes, vitamin E may indirectly reduce thrombotic risk and improve placental perfusion. Although clinical evidence regarding vitamin E supplementation in pregnancy remains variable, its mechanistic role in reducing oxidative stress provides a strong rationale for its involvement in modulating vascular complications (69–71).

Vitamin A further contributes to pregnancy outcomes through its role in cellular differentiation, immune regulation, and vascular development. The active metabolite retinoic acid regulates gene expression via nuclear receptors, influencing trophoblast differentiation, angiogenesis, and immune tolerance at the maternal–fetal interface. Adequate vitamin A levels are essential for proper spiral artery remodeling, a process that transforms maternal vessels into low-resistance channels capable of supporting the high metabolic demands of the developing fetus (72–75). Impairment of this remodeling process leads to placental hypoperfusion, which is a central feature of conditions such as preeclampsia and fetal growth restriction. In addition, vitamin A modulates immune responses, helping to maintain a balanced inflammatory environment that prevents excessive endothelial activation and coagulation. However, unlike other vitamins, vitamin A requires careful regulation, as both deficiency and excess are associated with adverse outcomes, including teratogenic effects at high levels (76, 77).

Collectively, fat-soluble vitamins act as critical regulators of the vascular, immune, and coagulation systems during pregnancy, influencing both the development and progression of prethrombotic conditions. Rather than functioning independently, these vitamins operate within an interconnected network that governs endothelial health, inflammatory balance, and placental perfusion. Their roles complement the direct anticoagulant effects of LMWH, suggesting that optimal maternal outcomes may depend on both pharmacological and nutritional modulation of thrombotic pathways. The integrated mechanisms and clinical implications of these vitamins are summarized in Table 2, highlighting their potential as adjunctive components in the management of pregnancy-associated thrombotic disorders.

**TABLE 2:** Mechanistic and clinical roles of fat-soluble vitamins in pregnancy-associated thrombotic conditions.

Vitamin	Primary biological targets	Key mechanisms	Impact on thrombosis and placenta	Clinical associations
Vitamin D	Endothelium, immune cells, trophoblasts	↓ TNF-α, ↓ IL-6, ↑ NO, ↓ PAI-1	↓ Endothelial dysfunction, ↑ fibrinolysis, ↑ placental perfusion	Preeclampsia, IUGR, miscarriage, preterm birth
Vitamin K	Coagulation factors (II, VII, IX, X), Protein C/S	γ-carboxylation of clotting proteins	Maintains coagulation balance (prevents bleeding + dysregulated clotting)	VK deficiency bleeding (maternal/neonatal)
Vitamin E	Lipid membranes, platelets, endothelium	Antioxidant (↓ ROS), ↓ platelet aggregation	↓ Oxidative endothelial injury →↓ prothrombotic signaling	Preeclampsia, vascular dysfunction
Vitamin A	Trophoblasts, vasculature, immune system	↑ Trophoblast invasion, angiogenesis, immune regulation	Prevents placental hypoperfusion →↓ microthrombosis	IUGR, placental insufficiency

## 5 Synergistic and complementary roles of LMWH and fat-soluble vitamins

The management of pregnancy-associated prethrombotic conditions has historically relied on anticoagulation, with low molecular weight heparin (LMWH) representing the gold standard for both prophylaxis and treatment of venous thromboembolism (VTE). However, increasing evidence indicates that thrombosis in pregnancy is not solely a disorder of coagulation, but rather a multifactorial process involving endothelial dysfunction, inflammation, oxidative stress, and abnormal placental development. These interconnected pathways form the basis of “immunothrombosis,” a concept that integrates immune activation with coagulation processes. In this broader context, fat-soluble vitamins—including vitamins D, K, E, and A—emerge as key modulators of the vascular and immune environment, providing complementary mechanisms that extend beyond the direct anticoagulant action of LMWH (78–80).

At the molecular level, LMWH primarily exerts its therapeutic effect by binding to antithrombin and selectively inhibiting factor Xa, thereby reducing thrombin generation and fibrin clot formation. This mechanism effectively limits clot propagation but does not directly address upstream drivers such as endothelial activation, cytokine signaling, or oxidative stress. In contrast, vitamin D has been shown to regulate inflammatory pathways and endothelial function by reducing proinflammatory cytokines and modulating fibrinolytic balance through suppression of plasminogen activator inhibitor-1 (81). These effects are particularly relevant in pregnancy, where increased PAI-1 and reduced fibrinolysis contribute to a prothrombotic state. Similarly, vitamin E mitigates oxidative stress by neutralizing reactive oxygen species, thereby preventing endothelial injury and platelet activation, both of which are key triggers of coagulation. Vitamin A contributes by supporting trophoblast invasion and vascular remodeling, addressing structural abnormalities that lead to placental hypoperfusion and secondary microthrombosis. Meanwhile, vitamin K ensures proper activation of both procoagulant and anticoagulant proteins, maintaining the balance of hemostasis in a system already shifted toward coagulation during pregnancy (82, 83).

Although both LMWH and fat-soluble vitamins have individually demonstrated potential benefits in pregnancy-associated thrombotic and placental disorders, direct clinical evidence evaluating their combined use remains limited. Most available evidence originates from studies investigating each intervention separately, including randomized controlled trials supporting LMWH in selected high-risk pregnancies and observational or interventional studies evaluating the effects of fat-soluble vitamin supplementation on inflammation, oxidative stress, endothelial function, and placental health. Consequently, the proposed combined approach should currently be regarded as a conceptual model supported by complementary biological mechanisms rather than an established therapeutic strategy.

The integration of these pathways suggests a synergistic model in which LMWH acts as a downstream inhibitor of clot formation, while fat-soluble vitamins function as upstream regulators that reduce the stimuli for coagulation activation. This dual-level intervention is particularly important in pregnancy, where thrombotic complications often originate from placental dysfunction and systemic inflammation rather than isolated clotting abnormalities. For example, placental microthrombosis and impaired spiral artery remodeling are key features of preeclampsia and fetal growth restriction. While LMWH can prevent further clot formation within placental vessels, vitamins such as D and A may improve placental vascular development and immune tolerance, thereby addressing the root causes of these conditions. This complementary action may enhance overall therapeutic efficacy by simultaneously targeting both cause and consequence (6, 84, 85).

From a clinical perspective, several studies support the independent benefits of LMWH and micronutrient status in high-risk pregnancies. LMWH has been shown to reduce VTE incidence and improve outcomes in women with thrombophilia or prior thrombotic events, with anticoagulation significantly lowering recurrence rates. In addition, LMWH use in conditions such as polycystic ovary syndrome (PCOS) has been associated with a marked reduction in early pregnancy loss, suggesting a beneficial effect on implantation and early placental development (86, 87). Concurrently, vitamin D deficiency has been consistently associated with increased risks of preeclampsia, intrauterine growth restriction, and miscarriage, reinforcing its role in vascular and immune regulation. Although direct interventional studies combining LMWH with vitamin supplementation remain limited, the convergence of mechanistic and clinical data provides a strong rationale for combined therapeutic approaches. A clinically integrated view of LMWH therapy and fat-soluble vitamin modulation in pregnancy-associated thrombotic conditions is summarized in Table 3, highlighting their complementary roles in targeting both coagulation and upstream pathological drivers (85, 88).

**TABLE 3:** Integrated clinical view of LMWH and fat-soluble vitamins in pregnancy-associated thrombotic conditions.

Clinical scenario	Pathophysiology	LMWH role (clinical evidence)	Vitamin role (clinical evidence)	Potential combined benefit
Venous thromboembolism (VTE)	Hypercoagulability, venous stasis, ↓ fibrinolysis	First-line anticoagulant; reduces VTE risk; safe (no placental transfer)	Vitamin D deficiency linked to higher thrombotic and inflammatory risk	LMWH prevents clot formation + Vitamin D reduces prothrombotic inflammation
Postpartum thrombosis	Peak hypercoagulability + endothelial injury	Continued LMWH reduces recurrence risk postpartum	Antioxidant vitamins (E, A) may reduce oxidative vascular damage	Combined vascular protection during highest-risk window
Preeclampsia	Endothelial dysfunction, inflammation, placental ischemia	Used in high-risk patients (improves placental perfusion in some studies)	Vitamin D deficiency strongly associated with preeclampsia; endothelial dysfunction central mechanism	LMWH improves microcirculation + Vitamin D improves immune/endothelial balance
Recurrent pregnancy loss (RPL)	Thrombophilia, placental microthrombosis	LMWH reduces early pregnancy loss (5.3% vs. 26.5%) (89, 90)	Vitamin D supports immune tolerance and placental function	Improved implantation + reduced placental thrombosis
Placental insufficiency/IUGR	Poor trophoblast invasion, microvascular thrombosis	Enhances placental blood flow (clinical observations)	Vitamin A and D regulate placental development and angiogenesis	Structural + functional placental improvement
Hemostatic imbalance	Increased clotting factors, reduced anticoagulants	Direct inhibition of Factor Xa and thrombin generation	Vitamin K regulates coagulation proteins (prothrombin activation)	Balanced coagulation system (avoid over/under clotting)
Inflammation-driven thrombosis (Immunothrombosis)	Cytokine activation, endothelial injury	Indirect anti-inflammatory effects	Vitamin D reduces cytokines (TNF-α, IL-6 pathways)	Dual suppression of inflammation-triggered clotting
Oxidative stress–mediated vascular injury	ROS → endothelial damage → clot initiation	No direct antioxidant effect	Vitamin E reduces oxidative stress (lipid peroxidation)	Protects vascular integrity + reduces clot triggers

Importantly, the postpartum period represents a particularly critical window where this synergy may be most beneficial. The risk of VTE increases dramatically after delivery, reaching up to 20-fold higher than in nonpregnant women. During this period, endothelial injury, inflammation, and hemodynamic changes further amplify thrombotic risk. While LMWH remains the primary prophylactic strategy, optimizing vitamin status may enhance vascular recovery and reduce residual inflammatory and oxidative stress, thereby contributing to a more comprehensive protective effect. Despite these promising insights, several limitations must be acknowledged. The heterogeneity of clinical studies, variability in dosing and supplementation protocols, and differences in baseline nutritional status complicate direct comparisons and limit definitive conclusions. Furthermore, excessive intake of certain fat-soluble vitamins, particularly vitamin A, carries risks of toxicity and teratogenicity, necessitating careful monitoring. Future research should focus on well-designed randomized controlled trials to evaluate the efficacy of combined LMWH and vitamin-based interventions, as well as the development of biomarker-driven strategies to personalize therapy.

Despite the strong mechanistic rationale, clinical validation of this complementary approach remains insufficient. At present, no randomized controlled trials have specifically evaluated the efficacy and safety of combining LMWH with fat-soluble vitamin supplementation for pregnancy-associated prethrombotic conditions. Existing evidence is largely derived from separate investigations of LMWH or individual vitamins, together with observational studies linking vitamin deficiencies to adverse pregnancy outcomes. Therefore, the potential clinical benefits of this combined strategy remain hypothetical and should be confirmed through well-designed prospective clinical trials before routine implementation can be recommended.

## 6 Clinical evidence and safety considerations

LMWH is an established anticoagulant for the prevention and treatment of pregnancy-associated thromboembolic complications. In contrast, clinical evidence supporting the combined use of LMWH with fat-soluble vitamins (vitamins A, D, E, and K) remains limited. While several mechanistic studies suggest that correction of vitamin deficiencies may improve endothelial function, immune regulation, oxidative stress, and placental homeostasis, direct clinical trials evaluating LMWH combined with fat-soluble vitamins are currently lacking. Consequently, any proposed synergy between these interventions should be regarded as biologically plausible rather than clinically established.

The safety and effectiveness of LMWH during pregnancy are supported by substantial clinical evidence. A systematic review including 2,777 pregnancies reported a venous thromboembolism rate of 0.86%, significant bleeding in 1.98% of pregnancies, no cases of heparin-induced thrombocytopenia, and a live birth rate of 94.7%, supporting LMWH as the standard anticoagulant for thromboprophylaxis and treatment in pregnancy (91). However, evidence supporting combination therapy in pregnancy primarily involves LMWH plus low-dose aspirin rather than vitamin supplementation. A recent meta-analysis of 14 studies demonstrated that LMWH combined with aspirin significantly increased live birth rates compared with aspirin or LMWH alone (OR 4.54, 95% CI 2.76–7.45) while reducing adverse pregnancy outcomes (OR 0.40, 95% CI 0.29–0.56) (92). Likewise, a meta-analysis of randomized controlled trials involving 1,758 women without inherited thrombophilia found that LMWH reduced the incidence of preeclampsia (RR 0.67, 95% CI 0.50–0.90), although this benefit was largely restricted to studies in which aspirin was administered concurrently (93). Conversely, LMWH has not demonstrated universal benefit across all obstetric conditions. In women with unexplained recurrent pregnancy loss, a multicenter randomized trial found no significant improvement in ongoing pregnancy or live birth rates with dalteparin compared with standard care, emphasizing that the clinical efficacy of LMWH remains indication-specific (94).

The potential contribution of fat-soluble vitamins should therefore be interpreted as supportive nutritional therapy rather than a proven anticoagulant strategy. Vitamin supplementation should primarily aim to correct documented deficiencies because excessive intake of fat-soluble vitamins may result in toxicity due to tissue accumulation. Vitamin A deserves particular attention, as excessive exposure during the first 60 days after conception has been associated with teratogenic risk. Recent nutritional modeling suggests that prenatal supplementation should provide approximately 198 μg retinol activity equivalents (RAE) of total vitamin A while limiting preformed retinol to no more than 2,063 μg to minimize toxicity risk (95). Acute hypervitaminosis A has been reported after ingestion of at least 500,000 IU in adults (96, 97). Similarly, routine high-dose vitamin E supplementation is not supported by current evidence. Although approximately 19 mg (28.5 IU) appears sufficient to meet maternal requirements, randomized trials administering 200–800 IU/day, frequently in combination with vitamin C, have not consistently reduced hypertensive disorders of pregnancy and have been associated with increased rates of abdominal pain, antihypertensive treatment, hospital admission for hypertension, fetal loss or perinatal death, and preterm prelabor rupture of membranes (83). Vitamin K also requires careful consideration because of its central role in coagulation biology. Protein C is a vitamin K-dependent endogenous anticoagulant, and vitamin K antagonists remain the standard long-term anticoagulant therapy for thrombotic antiphospholipid syndrome (APS) outside pregnancy, highlighting the need for caution when discussing vitamin K supplementation alongside anticoagulant therapy (98–100).

Clinical management should also be individualized according to underlying thrombotic risk. In obstetric APS, current guidelines recommend low-dose aspirin before conception followed by prophylactic LMWH after confirmation of pregnancy as standard care (101). Among women with APS and concomitant inherited thrombophilia, a cohort study involving 328 women and 1,332 pregnancies reported that inherited thrombophilia was present in approximately 14% of patients but was not associated with worse maternal or fetal outcomes when guideline-directed treatment was provided (102). Patients with hereditary antithrombin deficiency represent another distinct subgroup in whom anticoagulant intensity may substantially influence outcomes. In a retrospective study of 115 pregnancies, progressively higher LMWH doses were associated with lower venous thromboembolism risk, and administration of peripartum antithrombin concentrate reduced thromboembolic events from 25 to 1.5%, although hemorrhage exceeding 1,000 mL occurred in approximately 11% of term deliveries (103). In women without inherited thrombophilia, current evidence suggests that LMWH may reduce preeclampsia primarily in carefully selected high-risk populations, particularly when initiated early in pregnancy and combined with aspirin, whereas routine administration remains controversial (93, 104).

The postpartum period represents another critical phase requiring individualized thromboprophylaxis. Venous thromboembolism risk increases approximately 15–35-fold after delivery, although this risk declines substantially after the third postpartum week (105, 106). Current guidelines and clinician surveys continue to support 6 weeks of postpartum LMWH prophylaxis for women with previous venous thromboembolism or high-risk thrombophilia, despite limited high-quality evidence supporting the optimal duration (98, 105). The Highlow randomized trial involving 1,110 women demonstrated no significant difference between intermediate-dose and low-dose LMWH throughout pregnancy and 6 weeks postpartum, with recurrent venous thromboembolism rates of 2 and 3%, respectively, and identical major bleeding rates of 4% (107). Likewise, the LEAP pilot randomized trial enrolled only 30 participants, observed no thromboembolic events, and reported one delayed postpartum hemorrhage, illustrating the persistent uncertainty surrounding optimal postpartum anticoagulation strategies (106). Pharmacovigilance analyses have additionally identified pregnancy-specific safety signals, including injection-site hematoma, prolonged bleeding time, uterine hematoma, and peripartum hemorrhage among LMWH users, although these observational findings cannot establish causality (108).

## 7 Future perspectives

Despite the strong mechanistic rationale supporting the complementary roles of LMWH and fat-soluble vitamins in pregnancy-associated prethrombotic conditions, current evidence remains largely indirect, and their combined therapeutic application has not yet been validated in well-designed clinical trials. Future research should therefore move beyond mechanistic studies toward prospective investigations that establish the clinical efficacy, safety, and optimal implementation of this conceptual therapeutic approach. A major priority will be the identification of reliable biomarkers that enable personalized treatment strategies. Integrating biomarkers such as serum vitamin D and vitamin K status, inflammatory cytokines (e.g., IL-6, TNF-α), endothelial dysfunction markers, angiogenic factors (e.g., sFlt-1/PlGF ratio), oxidative stress indicators, and coagulation parameters (e.g., D-dimer, thrombin generation, PAI-1) may improve risk stratification, guide nutritional supplementation, and optimize anticoagulation therapy throughout pregnancy. Future clinical trials should also adopt risk-stratified study designs, evaluating whether selected patient populations—including women with inherited thrombophilia, antiphospholipid syndrome, recurrent pregnancy loss, preeclampsia, placental insufficiency, polycystic ovary syndrome, or a high risk of postpartum thrombosis—derive differential benefit from combining LMWH with targeted correction of vitamin deficiencies. Stratification according to gestational age, baseline nutritional status, and thrombotic risk may further facilitate precision medicine approaches. In parallel, randomized controlled trials are needed to establish the optimal dosage, timing, and duration of vitamin supplementation when used alongside LMWH, while carefully monitoring maternal and fetal safety. Particular attention should be given to the safe administration of fat-soluble vitamins, especially vitamin A and vitamin K, because excessive supplementation may result in toxicity or influence coagulation homeostasis. Standardized protocols for laboratory monitoring and dose adjustment should therefore be incorporated into future studies.

Finally, future research should aim to establish an integrated clinical pathway that combines anticoagulation therapy with evidence-based nutritional assessment and intervention. Such multidisciplinary strategies may facilitate individualized management of pregnancy-associated prethrombotic conditions by simultaneously addressing coagulation abnormalities, endothelial dysfunction, inflammation, oxidative stress, and placental vascular health. Although this integrated approach remains a conceptual model at present, continued advances in biomarker-guided therapy and well-designed clinical trials may ultimately support its translation into routine obstetric practice.

## Data Availability

The data that support the findings of this study are available from the corresponding author upon reasonable request.
